# Association of Primary Care Access with Health-Related ChatGPT Use: A National Cross-Sectional Survey

**DOI:** 10.1007/s11606-025-09406-9

**Published:** 2025-02-10

**Authors:** Oluwatobiloba Ayo-Ajibola, Catherine Julien, Matthew E. Lin, Jeffrey Riddell, Naihua Duan, Richard L. Kravitz

**Affiliations:** 1 Keck School of Medicine of the University of Southern California, Los Angeles, CA USA; 2 Department of Head and Neck Surgery, David Geffen School of Medicine at University of California Los Angeles, Los Angeles, CA USA; 3 Department of Emergency Medicine, Keck School of Medicine of the University of Southern California, Los Angeles, CA USA; 4 Division of Mental Health Data Science, Department of Psychiatry, Columbia University, New York, NY USA; 5 Department of General Medicine, Geriatrics, and Bioethics, University of California, Davis, Sacramento, CA USA

**Keywords:** artificial intelligence, chatGPT, patient information seeking, online health information, primary care

## Abstract

**Background:**

ChatGPT has quickly gained popularity as a source of online health information (OHI). However, it is unclear how having a usual source of primary care (USPC) is related to OHI-seeking.

**Objective:**

Explore how having a USPC and other characteristics thought to affect access-to-care influence the use of ChatGPT and other OHI forms.

**Design:**

Cross-sectional national survey.

**Participants:**

Adult members of ResearchMatch, a non-profit affiliate of the National Institutes of Health, between June and August 2023.

**Main Measures:**

The survey evaluated demographics, health characteristics, and OHI-seeking behaviors, including ChatGPT usage. OHI sources were categorized as “passive” (Google, Wikipedia, WebMD) and “interactive” (forums, Q&A sites, ChatGPT). Descriptive statistics, t-tests, and chi-square tests compared users by USPC status. Multiple logistic regression estimated adjusted effects on ChatGPT use.

**Key Results:**

Of 21,499 adults invited to participate in the survey, 2406 (11.2%) responded. Among respondents, 56% reported having a USPC. Those with a USPC, compared to those without, were older, spoke English as their primary language, had higher income, and had more formal education (all p<.001). Participants with a USPC were more likely to use passive OHI (OR 2.46, 95% CI 1.55–3.90, p<.001) and less likely to use interactive OHI (OR 0.73, 95% CI 0.60–0.89, p=.002) or ChatGPT (OR 0.56, 95% CI 0.44–0.71, p<.001). Age over 50 (OR 0.11, 95% CI 0.06–0.20, p<.001), non-White race (OR 0.51, 95% CI 0.38–0.70, p<.001), very good or better health (OR 0.71, 95% CI 0.55–0.92, p=.009), and college education (OR 0.61, 95% CI 0.39–0.97, p=.035) were inversely related to ChatGPT use.

**Conclusions:**

In this national survey of patients participating in a clinical research matching service, those with regular primary care access relied less on ChatGPT, suggesting that a personal primary care relationship may attenuate the need or motivation to use AI-derived OHI.

**Supplementary Information:**

The online version contains supplementary material available at 10.1007/s11606-025-09406-9.

## INTRODUCTION

Internet use has become integral to personal health management in the USA. For example, a 2022 study found that over 58% of US adults had sought health information online.^1^ As electronic information sources proliferate, the Internet is increasingly patients’ first point of contact for health concerns, sometimes displacing consultation with health care professionals.^2^ Patients often consult the internet for reassurance, second opinions, or extra context regarding a diagnosis, particularly when they experience challenges consulting healthcare providers (HCPs) directly.^3^

Within 2 months of its online public release in November 2022, the artificially intelligent (AI) chatbot ChatGPT gained 100 million monthly users, outpacing the adoption rates of both TikTok and Instagram.^4^ ChatGPT is a large language AI model trained on large quantities of textual data and refined by reinforcement learning with human feedback. This yields conversational chatbot responses to text input and enables further personalization of outputs based on user interaction, often with surprisingly high yet inconsistent accuracy across a range of health conditions.^5–8,10^

ChatGPT has become a major online health information (OHI) resource, yet patterns of use remain inadequately explored.^9^ In particular, the question of whether access to continuity-based versus episodic care alters the likelihood of patients seeking medical advice through ChatGPT has both clinical and policy implications.

Prior studies have found that patients with difficulties accessing primary care are more likely to consult the Internet for health information than those with easy access.^11,12^ Having a usual source of primary care (USPC) has in turn been associated with White race, female gender, older age, higher income, and higher levels of education.^13–16^ Patients cite difficulties accessing a USPC as a leading reason for visiting emergency departments for non-acute conditions, leading to potentially inappropriate utilization.^17–19^ However, little is known regarding the relationship between access to a USPC and patients’ inclinations to consult more interactive sources of OHI, such as ChatGPT, online forums, or Q&A sites like Quora, which require greater engagement from the information seeker. These “interactive sources” are distinguished from “passive sources,” like Google, Wikipedia, and WebMD, which dispense health advice by topic and are potentially less likely to return personally relevant information, with user experiences heavily influenced by search skills and health literacy.

To understand patterns of OHI use with a special emphasis on ChatGPT, we conducted an online survey in mid-2023.^20^ In earlier work, we found that ChatGPT users tended to be younger and have lower educational attainment than non-users. Many respondents reported that they consider ChatGPT’s information at least as useful as other OHI (87.7%) and the information provided by their doctor (81.0%). Regular access to a USPC may reduce patients’ reliance on ChatGPT by offering trusted human guidance on health-related matters. Alternatively, use may be increased as patients look to verify or supplement physician-supplied information. This study focuses on how access to a USPC relates to patient use of ChatGPT for health-related inquiries.

## METHODS

The University of Southern California Institutional Review Board approved this cross-sectional survey (UP# UP-23-00390-AM005I). Before completing the survey, participants were informed of the aim to measure OHI use and the various privacy and confidentiality protections, including digital and physical data safeguards. The message also emphasized that participants’ participation in the 10-min survey was voluntary and could not be linked to their identity.

### Model Adaptation and Questionnaire Creation

Given the recent release of ChatGPT, we created a novel survey instrument to assess patient experiences with ChatGPT (available in Appendix A). We drew upon multiple established approaches, including the Health Beliefs Model (HBM), which we applied to identify factors associated with health-related behaviors (Appendix B).^21^ The HBM posits that perceived susceptibility to illness, severity, self-efficacy, and anticipated barriers and benefits influence health behaviors. Such behaviors can be prompted by “cues to action” from events and peers. More details on our survey approach and the parent studies that inspired this work are in the first study our team published.^20^

### Study Measures

Our REDCap survey captured demographic details, including age, race, estimated total annual household income, highest level of education, and preferred language. Due to the inadvertent omission of a question on gender from early survey rounds, we used the final survey round (*n*=137) to obtain an estimate of the gender distribution of the full sample. We recorded participant access to community healthcare, further stratified to include a doctor’s office, urgent care or clinic, hospital ED, VA medical center, some other place, or no one place.^22,23^ USPCs included doctor’s offices, community clinics, and VA medical centers. Respondents provided self-perceived health statuses and health literacy levels via elements from the eHealth Literacy Scale.^3,24,25^

The survey assessed OHI use by asking if respondents consulted various websites, including passive sources like search engines and online health sites (e.g., WebMD, MedlinePlus, Mayo Clinic), and interactive sources like forums (e.g., Reddit, Facebook groups), Q&A sites, and ChatGPT. If respondents reported never seeking health information online, the survey ended there. Additional questions were also only presented if they selected ChatGPT as a source.

The survey examined respondents’ use of ChatGPT for both non-health-related and health-related purposes. Overall, we aimed to model regular OHI use, reflected in the population; in the preceding 6 months, 930 (85.7%) of participants with a USPC and 1039 (86.8%) without reported OHI use at least once a month (Question 19, Appendix A).

Respondents rated the ease of using ChatGPT for OHI, and the comprehensibility and relevance of the provided information, via 5-point scales from strongly agree (1) to strongly disagree (5).^26^ Ratings were summed and averaged to create a composite user experience score (moderate internal reliability via Cronbach Alpha of 0.773*)*.^26^ Participants also reported whether they shared ChatGPT findings with their doctor or planned to.

### Pilot Testing

Following multiple internal revisions, a pilot test was conducted to gather feedback on survey experience from five physicians, three medical students, and seven non-medical community members from various sociodemographic backgrounds. We altered the survey based on written and verbal feedback from community members, adding a question about rationale for ChatGPT OHI use, a “select all that apply” option for behaviors, and descriptive headings before each subsection to prime respondents for new topics.

### Survey Population

We collected survey responses via ResearchMatch, an online research participant recruitment registry operated by Vanderbilt University Medical Center and overseen by the U.S. National Institutes of Health that enables patients of all ages, ethnicities, and health statuses within the USA and Puerto Rico to participate in clinical research studies.^27^ ResearchMatch patients are invited to register by participating healthcare organizations. Researchers can contact registered participants with opportunities to join studies. ResearchMatch members are mostly Caucasian (71.6%) or African American (10.7%) and female (64%) with the majority denying medication use or chronic medical conditions (58.9% and 72.2%, respectively). Although our goal was to recruit participants with varied levels of healthcare access, a potential confound is that those enrolled in ResearchMatch may possess above-average health literacy and USPC access, as the platform requires enrollment during a visit to a participating healthcare center.

Recruitment was restricted to adults over 18 years in all 50 states. Since the ResearchMatch platform caps study invitations at 1500 unique participants per outreach cycle, we sent 11 batches of 1000–1499 invitations between June 10 and August 10, 2023, aiming for a final sample size of at least 2000.

### Survey Administration

We administered the survey via ResearchMatch to consenting adults between June 10th and August 10th, 2023. Our sole point of contact with participants occurred over the online ResearchMatch platform via a short message about the study’s goals, which included a link to the informed consent document. After providing consent and clicking the link to access the survey, participants were presented with the survey. To reduce item-missingness, participants had to complete all items on a page before moving forward and could not return to previous questions. However, missing responses could still occur due to incomplete survey completion. No identifying data for the respondents were retained. To guard against bot responses, we employed ReCAPTCHA technology. To optimize the response rate and minimize the number of incomplete responses, participants were incentivized by a raffle for two $50 gift cards with entry coordinated by a separate Qualtrics survey.

### Data Analysis

Using Stata Statistical Software: Release 18 (College Station, TX: StataCorp LLC), we characterized the sample using descriptive statistics and assessed differences between respondents with and without a USPC using Pearson’s chi-square tests and two-sided *t*-tests with a significance threshold of 0.05. We employed simple and multiple logistic regression models for factors associated with passive OHI, interactive OHI (including ChatGPT), and ChatGPT based on a literature review of factors traditionally associated with uptake of OHI such as race, income, literacy, and education. Incomplete survey responses were omitted from individual analyses where applicable without additional manipulation or sample weighting.

## RESULTS

### Cohort Composition (Table 1)

**Table 1:** Sample Demographic Characteristics

Characteristic, % (*n*)	All respondents (*n* = 2406)
Age (years)	
18–35	58.7 (1145)
36–49	23.9 (467)
50+	17.4 (339)
Race	
White	76.2 (1834)
Non-White	19.5 (486)
Preferred language	
English	72.4 (1742)
Non-English	23.3 (560)
Annual household income	
$49,999 or less	26.8 (644)
$50,000 to $74,999	23.6 (567)
$75,000 to $99,999	22.1 (531)
$100,000+	23.3 (560)
Education level	
High school or less	6.4 (153)
Some college	28.9 (695)
College or higher	60.4 (1454)
Health rating	
Good to poor	62.8 (1510)
Very good to excellent	32.9 (791)
Average eLiteracy Score—Mean (SD) Cronbach Alpha: 0.773*	3.81 (0.69)

Of the 21,499 ResearchMatch members invited to join the study, 2406 participated, for a response rate of 11.2%. Just over half (56%) reported having a USPC. The majority were 18–35 years old (59%), female (second sample, 74%), White (76%), spoke English (72%), and/or had graduated from college (60%); the median household income was $74,999. Most rated their health as good to poor (63%) (vs. very good to excellent). The average eHealth literacy score (1–5 scale, 5=greater literacy) was 3.81 (SD 0.69).

### Who Has a USPC? (Table 2)

**Table 2:** Respondent Demographic Characteristics by USPC Status

Characteristic, *n* (%)	USPC (*n* = 1345)	Non-USPC (*n* = 1061)	*p*-value
Age (years)			<0.001
18–35	468 (42.7)	677 (51.7)	
36–49	232 (21.2)	235 (17.9)	
At least 50	256 (23.4)	83 (6.3)	
Race			<0.001
White	843 (76.9)	991 (75.7)	
Non-White	253 (23.1)	215 (16.4)	
Preferred language			<0.001
English	1080 (98.5)	1164 (88.9)	
Non-English	16 (1.5)	42 (3.2)	
Annual household income			<0.001
$49,999 and under	304 (27.7)	340 (26.0)	
$50,000 to $74,999	241 (22.0)	326 (24.9)	
$75,000 to $99,999	222 (20.3)	309 (23.6)	
At least $100,000	329 (30.0)	231 (17.6)	
Education level			<0.001
High school or less	54 (4.9)	99 (7.6)	
Some college	252 (23.0)	443 (33.8)	
College or higher	790 (72.1)	664 (50.7)	
Health rating			<0.001
Good to poor	676 (61.7)	834 (66.7)	
Very good to excellent	420 (38.3)	371 (28.3)	
Average eLiteracy Score—Mean (SD) Cronbach Alpha: 0.773*	3.94 (0.02)	3.70 (0.02)	<0.001

^*^Generated score is an average of scores from 3 questions pertaining to ease of use, understanding, and relevance of ChatGPT OHI, measured from Strongly Disagree (1) to Strongly Agree (5); (Cronbach α=0.645).

Respondents with a USPC were significantly older and more likely than those without a USPC to prefer English for health communication, have higher income, and have more formal education (all *p*<.001). A greater proportion of non-White respondents were among the cohort with a USPC than the cohort without (23.1% vs. 16.4%, *p*<.001). Those with a USPC also had greater health literacy (3.94 vs. 3.70 on 1-5 scale, *p*<.001).

### How Does the Use of Passive OHI, Interactive OHI, and ChatGPT OHI Use Vary Across Different Sources of Usual Care? (Fig. 1)

**Figure 1:**
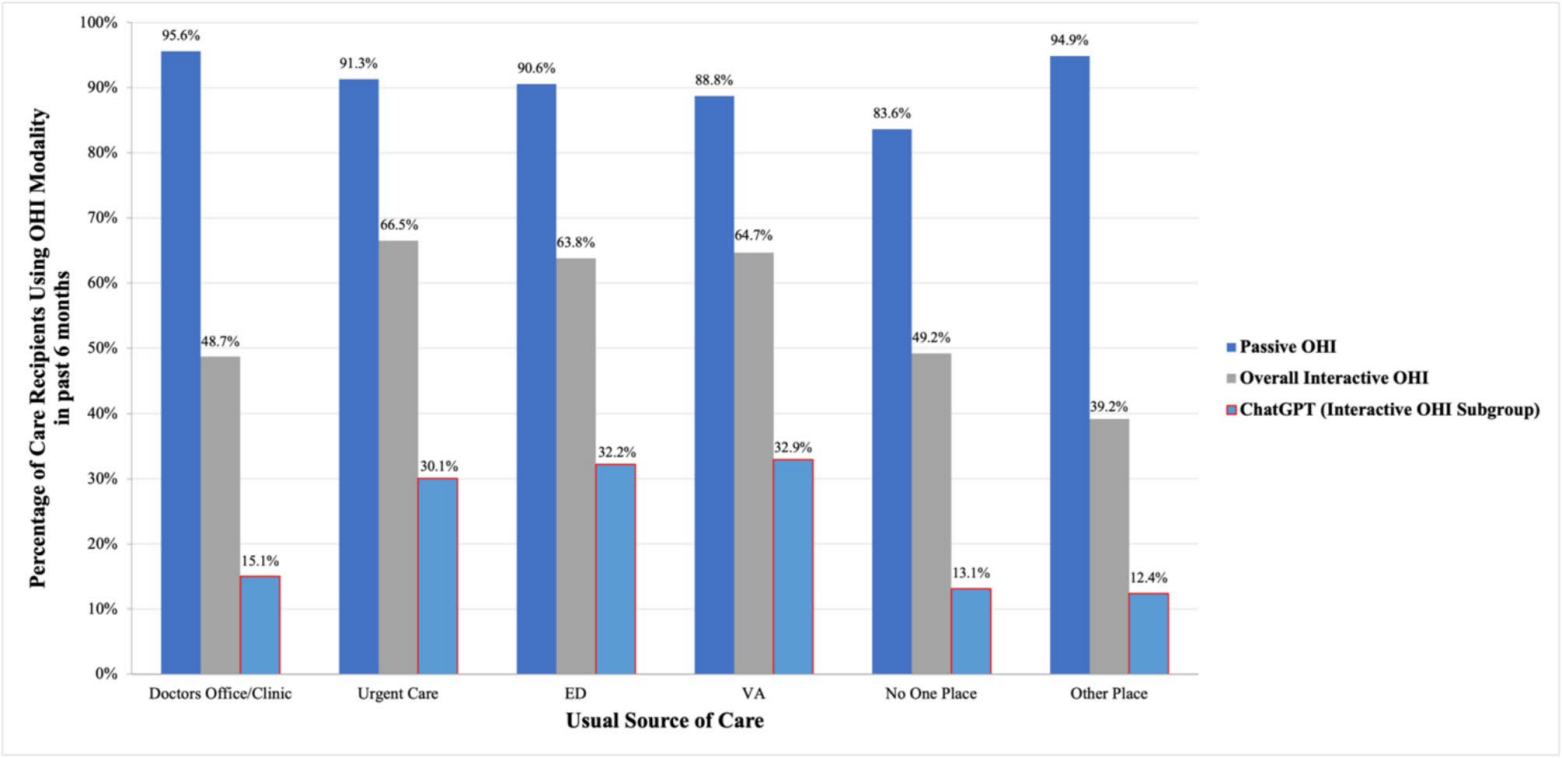
Use of OHI modalities stratified by usual source of care. Dark blue bars correspond to use of passive online health information, gray bars correspond to use of interactive health information (including ChatGPT), and light blue bars with red outlines correspond to use of ChatGPT specifically (a subgroup of interactive online health information). The percentages indicate the proportion of those receiving usual care from a specific source who endorsed use of a specified OHI modality during the last 6 months. These responses were obtained via a “select all that apply” question format, where respondents could choose from a list of several examples of passive and interactive online health information sources.

Figure 1 shows the prior 6-month prevalence of OHI use by USPC status. Passive OHI use ranged from 84% (no usual source of care) to 96% (doctor’s office or clinic). Interactive OHI use ranged from 39% (“other place”) to 67% (urgent care). Interactive OHI use among patients getting care in doctors’ offices and clinics was 49%. ChatGPT use followed similar patterns to those of interactive OHI, with the highest use among patients seeking care in urgent care, emergency, and VA settings (30 to 33%) and the lowest use among patients receiving regular care in a different setting than listed as an option (12%).

### How Does OHI Seeking Differ Between Patients With and Without a USPC? (Table 3)

**Table 3:** Respondent OHI Use Behaviors by USPC Status

Characteristic, *n* (%)	USPC (*n* = 1345)	Non-USPC (*n* = 1061)	*p*-value
OHI methodology			
Use of passive OHI (e.g. Google, Wikipedia, WebMD)	1269 (94.4)	868 (81.8)	<0.001
Use of active OHI (e.g., online forums, Q&A sites like Quora, ChatGPT)	695 (51.7)	591 (55.7)	0.049
Use of ChatGPT OHI	247 (18.4)	270 (25.5)	<0.001
Prior use of ChatGPT	214 (48.8)	225 (51.3)	0.137
Six month general OHI-seeking frequency			0.001
Weekly or more	653 (48.6)	530 (49.9)	
Monthly or Less	681 (50.6)	418 (39.4)	
Use frequency of ChatGPT OHI			0.302
Once a week or less	139 (57.7)	166 (62.2)	
2–3 times a week to daily	102 (42.3)	101 (37.8)	
Type of issue prompting engagement with ChatGPT			0.003
New health issue	45 (28.1)	50 (14.5)	
Long-standing health issue	46 (28.8)	95 (27.5)	
Both new and long-standing health issue	51 (31.9)	151 (43.8)	
General public health issue	17 (10.6)	48 (13.9)	
Other	1 (0.6)	1 (0.0)	
User experience scale (ease, understanding, relevance)—*Mean (SD) Cronbach's Alpha: 0.645*^*a*^	3.85 (0.66)	3.64 (0.68)	<0.001
Suspected inaccuracy	147 (63.4)	189 (71.9)	0.043
Presented information to doctor	153 (61.9)	196 (72.6)	0.010
Consulted doctor for clarification	114 (40.0)	108 (46.2)	0.158

Respondents with a USPC were more likely than those without a USPC to report using passive OHI within the past 6 months (94% vs. 82%, *p*<.001) but less likely to use interactive OHI (52% vs. 56%, *p*=.049) and ChatGPT specifically (18% vs. 26%, *p*<.001). However, they were similarly likely to report prior use of ChatGPT for non-OHI purposes (49% vs. 51%, *p*=.14). Users with a USPC were more likely than those without to consult ChatGPT for new health issues only (28% vs. 14%), and similarly likely to use it for long-standing health issues only (29% vs. 28%), while users without a USPC were more likely to consult the resource for both new and long-standing health issues (44% vs. 32%) (*p*=.003). Additionally, participants without a USPC were more likely than those with a USPC to use ChatGPT for general public health matters (14% vs. 11%, *p*=.003). Composite experience scores (understandability, ease of use, and relevance) were higher among respondents with a USPC than without (mean 3.85 vs 3.64, *p*<.001). Furthermore, those with a USPC were less likely to suspect ChatGPT inaccuracies (63% vs. 72%, *p*=0.043), and their inclination to discuss information they learned with their doctors was lower (62% vs 73%, *p*=.010).

### What Are the Adjusted Effects of Having a USPC on the Likelihood of Using Passive OHI, Interactive OHI, and ChatGPT? (Table 4)

**Table 4:** Multiple Logistic Regression of Factors Associated With ChatGPT Use

	ChatGPT use		
Characteristic	Odds ratio	95% CI	*p*-value
Continuity care access			
Non-USPC	ref		
USPC	0.56	0.44–0.71	**<0.001**
Age (years)			
18–35	ref		
36–49	0.79	0.61–1.03	0.083
At least 50	0.11	0.06–0.20	**<0.001**
Race			
White	ref		
Non-White	0.51	0.38–0.70	**<0.001**
Preferred language			
English	ref		
Non-English	1.86	0.89–3.90	0.100
Annual household income			
$49,999 and under	ref		
$50,000 to $74,999	1.21	0.90–1.63	0.21
$75,000 to $99,999	0.86	0.63–1.19	0.37
At least $100,000	0.73	0.52–1.04	0.081
Education level			
High school or less	ref		
Some college	1.13	0.72–1.77	0.603
College or higher	0.61	0.39–0.97	**0.035**
Health rating			
Good to poor	ref		
Very good to excellent	0.71	0.55–0.92	**0.009**
Average eLiteracy Score			
1 to <3	ref		
3 to <3.75	2.19	1.33–3.60	**0.002**
3.75 to <4.25	2.11	1.29–3.45	**0.003**
4.25 to 5	2.13	1.27–3.57	**0.004**

In a multiple logistic regression for ChatGPT OHI use controlling for age, race, preferred language, household income, education, health status, and health literacy, having a USPC was associated with lower adjusted odds of ChatGPT use during the past 6 months (OR 0.56, 95% CI 0.44–0.71, *p*<.001). Not surprisingly, age over 50 (OR 0.11, 0.06–0.20, *p*<.001), non-White race (OR 0.51, 0.38–0.70, *p*<.001), and very good or better health (OR 0.71, 0.55–0.92, *p*=0.009) were associated with lower odds of use. Contrary to expectations, having a four-year college degree was associated with lower odds of ChatGPT use (OR 0.61, 95% CI 0.39–0.97, *p*=.035).

Two logistic regressions analyzed passive and interactive OHI use (Appendix C). Having a USPC increased the odds of passive OHI use in the past 6 months (OR 2.46, 95% CI 1.55–3.90, *p*<.001). Older age (50+), non-White race, better health, and high health literacy were linked to higher passive OHI usage, while middle age (36–49) was linked to lower usage.

Conversely, having a USPC decreased the odds of interactive OHI use (OR 0.73, 95% CI 0.60–0.89, *p*=.002). Older age, non-White race, and better health were also associated with lower interactive OHI usage. Moderate to high eHealth Literacy scores correlated with increased odds for both interactive and passive OHI use.

## DISCUSSION

Over 40 million Americans either have no usual source of care or rely on settings designed to provide episodic care (emergency departments, urgent care centers). As shown in prior studies, impaired continuity of care raises susceptibility to poor health outcomes and increased healthcare utilization.^28–31^ Prior investigations have linked primary care access with increased utilization of OHI.^11,12^ However, none have explored the relationship with ChatGPT and other AI-derived health information. Our investigation is among the first to explore this relationship specifically for ChatGPT OHI. Given the growing role of interactive OHI in personal health management, it is important to understand the relationship between continuity-based healthcare and patients’ use of interactive OHI, including ChatGPT. ^20,32–35^

This study yielded three principal findings. First, in alignment with past work, primary care access was lower among non-English-speaking, younger, less educated, and lower-income patients.^16,36–38^ Our survey sample unexpectedly showed high rates of having a USPC among racial/ethnic minorities, seemingly contradicting the accepted understanding that minority identity is associated with poor USPC access.^16,17,35,37^ This discrepancy may stem from the fact that ResearchMatch features a predominantly White cohort and requires affiliation with a partnering healthcare system for enrollment, which may increase the likelihood of having a USPC. As shown in prior literature, participants with a USPC (who generally enjoyed higher incomes and greater educational attainment) had greater health literacy than those without.^24,39–41^

Second, participants with a USPC were more likely to seek passive OHI. Passive OHI sources often display information at reading levels far above the average American’s 5^th^-grade level.^42,43^ Greater use among those with USPC (even after adjustment for sociodemographic characteristics and health literacy) may be due to better access to clinicians who can explain the nuances of information from passive sources.

Third, those with a USPC showed decreased reliance on interactive OHI, especially ChatGPT. Interactive OHI involves interaction with other forum members, whose credibility may vary, or with ChatGPT, which is known for producing information “hallucinations,” leading to decreased accuracy.^44^ Reduced utilization of interactive OHI may be attributed to patient’s access to a human “interpreter,” in the form of their continuity-of-care clinician, who can explain complex health information and identify the information that is salient to the patient’s presentation. The result is a higher likelihood of patients receiving accurate and relevant health information.^45–47^ This is a function that those without a USPC may attempt to replace with ChatGPT. Our study found that respondents with a USPC were less likely to suspect inaccuracies in ChatGPT OHI, perhaps due to greater confidence in their ability to tease out needed information. They were also less likely to share their ChatGPT inquiries with their doctor, suggesting more direct reliance on physicians for health advice. These findings may also relate to their engagement with other OHI sources, higher eHealth literacy, and better overall health status, which could decrease reliance on novel forms of OHI when questions arise. Furthermore, trust in primary care physicians, strengthened through continuity of care and easier access to secure messaging portals, may enhance patient confidence and reduce perceived need for interactive OHI.^48–50^

### Limitations and Future Directions

Our study has limitations. First, the cross-sectional design precludes causal inference. Second, participant recruitment via ResearchMatch likely skews the sample towards more educated, health-literate, Internet-savvy individuals who use medical services. Using only a subset of the eHealth Literacy Scale decreased respondent burden but risks altering established psychometric properties and thus limits interpretability. Additionally, the low response rate affects generalizability. Despite these limitations, this study indicates the role of primary care in providing personalized health advice that is useful for all patients.

Future research should examine how satisfaction with current care affects the use of alternative OHI sources, irrespective of continuity care access. Health equity considerations necessitate understanding AI-derived OHI usage among underserved populations and those with chronic conditions. It is also crucial to investigate how clinicians and AI health information sources may substitute or complement each other. Understanding how patients choose these sources and how clinicians can guide them to suitable options remains a key challenge for future research.

## CONCLUSION

Our study found that access to a USPC is associated with a decreased likelihood of using ChatGPT for OHI. These results suggest a previously unheralded benefit of primary care in providing personalized counseling for health-related questions and reducing the need for quasi-personalized sources like ChatGPT.

## Supplementary Information

Below is the link to the electronic supplementary material.

Supplementary file1 (PDF 434 KB)

Supplementary file2 (PNG 102 KB)

Supplementary file3 (DOCX 22 KB)

## Data Availability

The datasets collected and analyzed during the current study are available from the first author on reasonable request (ayoajibo@usc.edu).

## Abbreviations

OHI: Online health information
HCP: Healthcare provider
AI: Artificial intelligence
