# Supplementary figures and images for: Association of Primary Care Access with Health-Related ChatGPT Use: A National Cross-Sectional Survey
Source: J Gen Intern Med. 2025 Feb 10;41(2):338–45. doi: 10.1007/s11606-025-09406-9 (PMC12894562; doi:10.1007/s11606-025-09406-9)

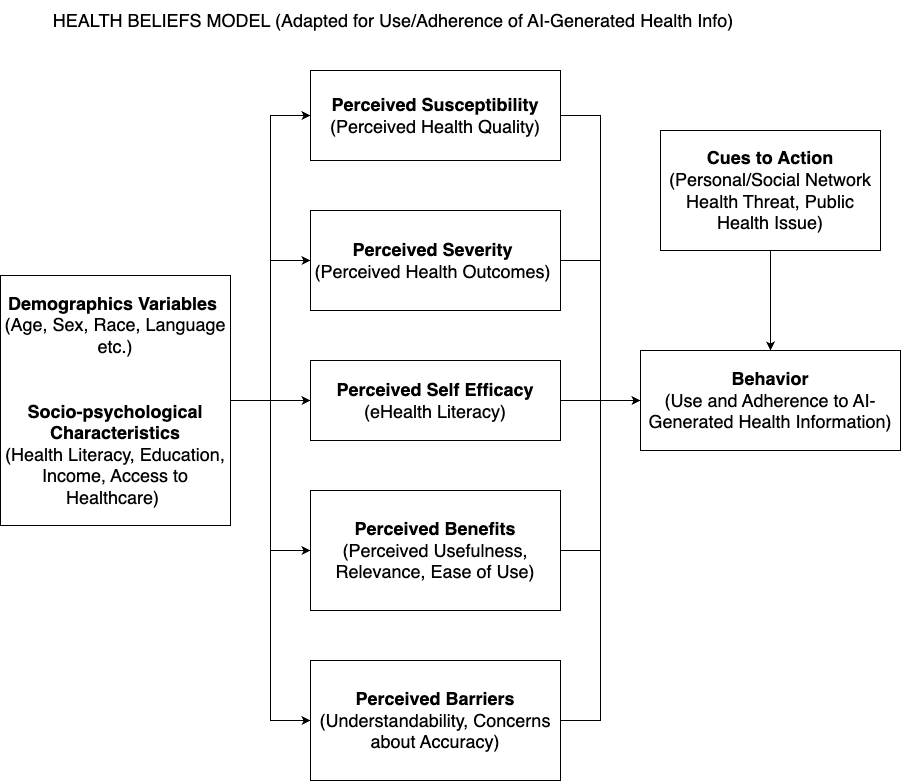

Supplement: Supplementary file 2 — Supplementary file2 (PNG 102 KB) [file 11606_2025_9406_MOESM2_ESM.png]
