# Supplementary material for: Association of Primary Care Access with Health-Related ChatGPT Use: A National Cross-Sectional Survey
Source: J Gen Intern Med. 2025 Feb 10;41(2):338–45. doi: 10.1007/s11606-025-09406-9 (PMC12894562; doi:10.1007/s11606-025-09406-9)
Supplement: Supplementary file 3 — Supplementary file3 (DOCX 22 KB) [file 11606_2025_9406_MOESM3_ESM.docx]

| **Appendix C: Multiple Logistic Regression of Factors Associated with OHI Use (TO BE PUBLISHED ONLINE AS SUPPLEMENTARY APPENDIX)** | | | | |  |  |
| --- | --- | --- | --- | --- | --- | --- |
|  | **Passive OHI use** | | | **Interactive OHI Use** | | |
| **Characteristic** | **Odds Ratio** | **95% CI** | **p-value** | **Odds Ratio** | **95% CI** | **p-value** |
| **Continuity Care Access** |  |  |  |  |  |  |
| Non-USPC | ref |  |  | ref |  |  |
| USPC | 2.46 | 1.55 - 3.90 | **<0.001** | 0.73 | 0.60 - 0.89 | **0.002** |
| **Age (years)** |  |  |  |  |  |  |
| 18-35 | ref |  |  | ref |  |  |
| 36-49 | 0.67 | 0.43 - 1.04 | **0.072** | 0.94 | 0.75 - 1.19 | 0.610 |
| At least 50 | 1.28 | 0.63 - 2.59 | 0.502 | 0.22 | 0.17 - 0.30 | **<0.001** |
| **Race** |  |  |  |  |  |  |
| White | ref |  |  | ref |  |  |
| Non-White | 1.10 | 0.65 - 1.86 | 0.723 | 0.77 | 0.61 - 0.98 | **0.037** |
| **Preferred Language** |  |  |  |  |  |  |
| English | ref |  |  | ref |  |  |
| Non-English | 0.47 | 0.17 - 1.30 | 0.143 | 1.10 | 0.55 - 2.17 | 0.794 |
| **Annual Household Income** |  |  |  |  |  |  |
| $49,999 and under | ref |  |  | ref |  |  |
| $50,000 to $74,999 | 0.67 | 0.38 - 1.19 | 0.170 | 0.81 | 0.62 - 1.05 | 0.115 |
| $75,000 to $99,999 | 0.47 | 0.26 - 0.82 | **0.009** | 1.06 | 0.81 - 1.41 | 0.661 |
| At least $100,000 | 0.70 | 0.36 - 1.33 | 0.270 | 0.94 | 0.71-1.24 | 0.677 |
| **Education Level** |  |  |  |  |  |  |
| High School or Less | ref |  |  | ref |  |  |
| Some College | 0.43 | 0.15 - 1.22 | 0.113 | 1.17 | 0.77 - 1.78 | 0.453 |
| College or Higher | 0.55 | 0.19 - 1.59 | 0.270 | 1.00 | 0.66 - 1.50 | 0.999 |
| **Health Rating** |  |  |  |  |  |  |
| Good to Poor | ref |  |  | ref |  |  |
| Very Good to Excellent | 1.41 | 0.88 - 2.24 | 0.153 | 0.58 | 0.47 - 0.71 | **<0.001** |
| **Average eLiteracy Score** |  |  |  |  |  |  |
| 1 to <3 | ref |  |  | ref |  |  |
| 3 to <3.75 | 0.99 | 0.51 - 1.91 | 0.967 | 2.11 | 1.41 - 3.16 | **<0.001** |
| 3.75 to <4.25 | 2.04 | 1.02 - 4.08 | **0.044** | 1.83 | 1.24 - 2.70 | **0.003** |
| 4.25 to 5 | 2.36 | 1.10 - 5.07 | **0.028** | 1.89 | 1.26 - 2.83 | **0.002** |
